# Secondary Metabolites Produced by Plant Growth-Promoting Bacterial Endophytes

**DOI:** 10.3390/microorganisms10102008

**Published:** 2022-10-11

**Authors:** Zareen Narayanan, Bernard R. Glick

**Affiliations:** 1 Division of Biological Sciences, School of STEM, University of Washington, Bothell, WA 98011, USA; 2 Department of Biology, University of Waterloo, Waterloo, ON N2L3G1, Canada

**Keywords:** therapeutic applications, secondary metabolites, plant growth promoting bacteria, endophytes, mechanisms

## Abstract

There is an increasing interest in the use of beneficial microorganisms as alternatives to chemically synthesized or plant-derived molecules to produce therapeutic agents. Bacterial endophytes are plant-associated microorganisms that can colonize different parts of living plants without causing any diseases. Diverse endophytic bacteria possess the ability to synthesize a wide range of secondary metabolites with unique chemical structures that have been exploited for their anti-microbial, antiviral, anti-cancer, and anti-inflammatory properties. Additionally, production of these bioactive compounds can also benefit the host plant as they may play a significant role in a plant’s interaction with the environment for adaptation and defense. As a result of their significant impact as curative compounds or as precursors to produce new drugs, the biotechnological possibilities of secondary metabolites derived from endophytic bacteria are immense.

## 1. Introduction

One of the rising concerns in the field of medicine is the rapid increase in drug resistance and new strains of virulent microorganisms [1,2]. To address this issue, there is a renewed interest in searching for novel chemical compounds from natural sources that represent sustainable and impactful means of finding new drugs. Endophytes are plant-associated microbes with rich species diversity and an extremely wide host range and are viewed as a promising source of natural products. Reportedly, every single plant studied to date is colonized by one or more types of endophytes [3]. Microbial endophytes form a symbiotic relationship with plants where the endophytes live inside the host plant without causing any apparent symptoms of disease. Bacterial endophytes have been isolated from all parts of plants including flowers, seeds, leaves, roots, and stems [4]. They produce primary and secondary metabolites that can greatly influence the host plant’s metabolism and produce various biological effects. For example, bacterial endophytes can promote host plant health, enhance growth and yield across plant species, and improve tolerance to various pathogens and environmental stresses [5,6,7,8,9,10,11].

In recent years, research on endophytes has moved from the traditional physiological and biochemical studies to cellular and molecular research, thus providing insights for the future commercial development of endophytes. Modern genomic tools have helped scientists understand biological activities during plant-endophyte interactions and many metabolomic studies have revealed that endophytes can act as reservoirs of novel bioactive secondary metabolites including alkaloids, benzopyrones, chinones, flavonoids, tetralones, xanthones, terpenoids, phenolic compounds, steroids, quinones, tannins and many other subclasses. These compounds represent a promising resource of novel natural products with significant biological important activities such as antimicrobial, anti-cancer, antioxidant, antiviral, and immunosuppressive activities [12]. Antimicrobial compounds may also be used as food preservatives among other biotechnological applications [13]. Furthermore, advances in chromatography and spectroscopy techniques have smoothened the path of rapid identification of known and unknown secondary metabolites [14,15].

In this review, we provide an overview of plant bacterial endophytes and the mechanisms that they use to facilitate plant growth along with a discussion of the different secondary metabolites synthesized by these microbes. The potential application of these secondary metabolites as anti-microbial, anti-cancer, anti-viral and anti-inflammatory agents is discussed.

## 2. Plant Bacterial Endophytes

### 2.1. An Overview of Plant Growth-Promoting Bacterial Endophytes

**Figure 1:**
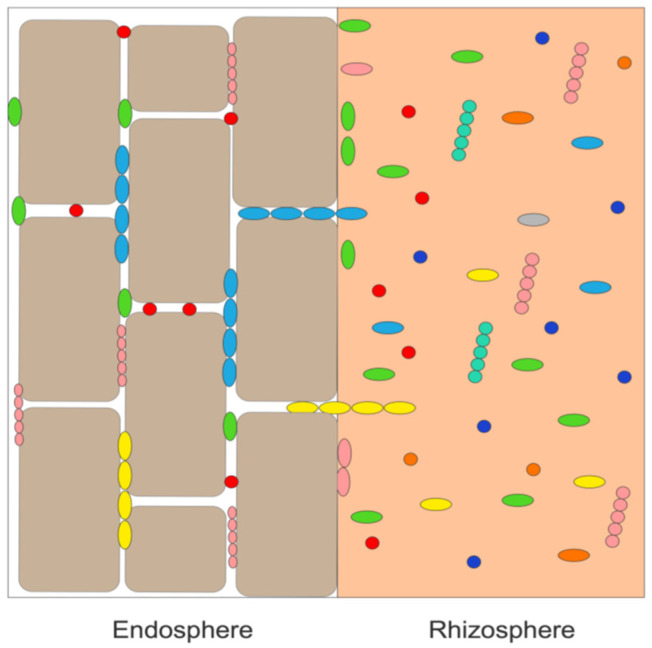
Schematic representation of various rhizosphere bacteria (as shown by different colored shapes) being taken up from the rhizosphere and localized intercellularly in the plant root endosphere.

Plant growth-promoting bacterial endophytes are plant growth-promoting bacteria (PGPB) that are typically present as free-living bacteria in the soil immediately around the plant’s roots (the rhizosphere). From the rhizosphere, these bacteria generally enter a plant through root wounds and cracks [16,17]. It has been estimated that nearly all the world’s ~300,00 plant species [18] contain several different endophytic bacteria (~10^5^ – 10^8^ cells per gram of plant tissue) as well as numerous fungal endophytes. Importantly, bacterial endophytes can grow inside plant tissues in a mutualistic relationship with the plant without harming or inhibiting the growth of the plant. As depicted schematically in Figure 1, endophytic bacteria are mostly found between plant cells (i.e., intercellularly), whereas fungal endophytes (not shown in this figure) are typically found inside of plant cells (i.e., intracellularly). Moreover, while endophytic PGPB are attracted to a specific plant’s root exudates [19,20] and enter the plant through the roots, many of these bacteria are motile and can travel through the plant to other tissues such as leaves and stems (where they are generally found in lower concentration than in the plant roots). In addition, various plant species and subspecies, plant organs and different stages of plant growth exude a different range of small organic molecules, and therefore, attract different bacteria [21]. Consequently, different tissues within the same plant may contain different groups of bacterial (and fungal) endophytes [22].

Plants benefit from the presence of endophytic PGPB because of the multiplicity of mechanisms that these organisms can use to facilitate plant growth (see Section 2.3, Section 2.4 and Section 2.5). Here, it is necessary to keep in mind that a single endophytic PGPB is likely to be endowed with several (but not all possible) mechanisms of plant growth promotion. However, a consortium of endophytic PGPB working together may be able to provide a wide range of activities that are important in stimulating plant growth and development [23,24]. Endophytic PGPB benefit from being inside the plant’s interior because of the advantages of this biological niche. Advantages of endophytic growth include protection from competing bacteria and fungi, a constant and reliable source of nutrition, and protection from exposure to a wide range of potentially deleterious environmental conditions, such as extremes of temperature and the presence of inhibitory chemicals in the soil. 

### 2.2. Isolation of Bacterial Endophytes 

Since endophytic PGPB have been found to colonize nearly all plant species [18,25] they can be isolated directly from various plant tissues. This is done by harvesting plant tissue (most commonly this is either roots, stems or leaves) and then sterilizing the outer surface of that tissue with a 10% bleach (sodium hypochlorite) solution and then with ~70% ethanol; surface sterilization of plant tissues is the most critical step in this process. Tween-20, Tween-80 and Triton X-100 have also been used to facilitate the surface sterilization process [26]. The surface-sterilized tissue is treated with sodium bicarbonate to inhibit fungal growth, then macerated, followed by removal of the solid material, and then dilutions of the plant sap are plated onto selective solid media, keeping in mind that a large portion (estimated to be >90%) of environmental bacterial samples are recalcitrant to growing in laboratory culture. The individual colonies that form on selective media are then characterized for a variety of traits. A variation of this approach includes collecting soil samples from a range of selected relevant environments and then planting sterilized seeds in those soil samples. Many of the endophytic PGPB that are present in the soil samples will be taken up into the roots (and perhaps into the shoots and leaves as well) of the growing plant and may be isolated as indicated above from specific pieces of plant tissue [27,28,29]. Initial characterization of bacterial endophytes typically includes sequencing the DNA of their 16S rRNA genes [30].

### 2.3. Endophytic PGPB Mechanisms That Directly Promote Plant Growth

Bacterial endophytes employ a wide range of mechanisms, following their interaction with plants, where they directly promote plant growth and development. Endophytic PGPB appear to use a similar, if not identical, repertoire of mechanisms to directly promote plant growth as rhizospheric PGPB [17,31,32,33,34,35]. These mechanisms include (but may not be limited to) the production of molecules involved in inorganic phosphate and potassium solubilization (e.g., various low molecular weight organic acids); synthesis of siderophores (chelating agents) that sequester iron from the soil and provide it to plants; synthesis of gibberelins and cytokinins (phytohormones that regulate various plant developmental processes); synthesis of auxins (such as indole 3-acetic acid, the most common auxin) which are phytohormones that promote plant cell elongation and proliferation; synthesis of the unusual and highly stable water-structuring sugar molecule trehalose which can help the plant to lower (overcome) salt and drought stress; the ability to fix atmospheric nitrogen into the ammonia which is necessary to synthesize proteins and nucleic acids; and synthesis of the enzyme 1-aminocyclopropane-1-caroxylate (ACC) deaminase which lowers plant ethylene levels, thereby decreasing the inhibitory effects of various abiotic stresses (see Section 2.5). There are often genes encoding the biosynthesis of plant hormones including auxin [36], cytokinin [37] and gibberellin [38] found within the microbiome of endophytic communities (although not necessarily within the same bacterium). While all these mechanisms may be involved in promoting plant growth and development, the synthesis of ACC deaminase is arguably the key mechanism in the promotion of plant growth by PGPB [39].

### 2.4. Endophytic PGPB Mechanisms That Indirectly Promote Plant Growth

The indirect promotion of plant growth occurs when a PGPB prevents or lessens plant growth inhibition that is caused by plant pathogens. These pathogens are most often fungi but also include some bacteria, insects, and nematodes. Some endophytic PGPB utilize (biocontrol) mechanisms that thwart the functioning of various phytopathogens. However, these endophytic PGPB do not necessarily stimulate the growth of the plant directly. These indirect mechanisms include the synthesis of (i) antibiotics, (ii) hydrogen cyanide, (iii) fungal cell wall hydrolyzing enzymes, (iv) siderophores (which deprive phytopathogens of sufficient iron for their proliferation), (v) phytopathogen inhibiting volatile organic compounds (VOCs), (vi) chemical compounds that induce systemic resistance (ISR) within target plants, and (vii) ACC deaminase (which lowers the plant’s level of growth inhibiting stress ethylene) [17]. 

Below are a few recent examples of endophytic PGPB indirectly promoting plant growth. (i) da Siveira et al. [40] isolated endophytic PGPB from the roots of sugarcane plants and found that several bacterial strains that produced siderophores, hydrogen cyanide, and VOCs inhibited the proliferation of the fungal phytopathogens *Bipolaris sacchari* and *Ceratocystis paradoxa*. (ii) Worsley et al. [41] reported isolating an endophytic strain of *Streptomyces* that demonstrated broad-spectrum antimicrobial activity and synthesized the compound 14-hydroxyisochainin which inhibited the proliferation of the pathogenic fungus, *Gaeumannomyces graminis* var. tritici (wheat take-all fungus). (iii) Gupta et al. [42] found a large decrease in the disease mortality of pea plants infected with the fungal phytopathogen *Fusarium oxysporum* when they were treated with a consortium of endophytic PGPB that produced VOCs and elicited ISR. (iv) Hamaoka et al. [43] noted that the endophytic PGPB *Bacillus velezensis* KOF112, originally isolated from Japanese wine grapes, inhibited the mycelial growth of the fungal phytopathogens *Botrytis cinerea*, *Colletotrichum gloeosporioides*, and *Phytophthora infestans* (where strain KOF112 synthesized antibiotics and elicited ISR in treated plants). (v) Uwaremwe et al. [44] discovered that an endophytic strain of suppressed root rot of Chinese wolfberry (*Lycium barbarum*) caused by *Fusarium oxysporum* functioned by modifying the amounts of various wolfberry rhizospheric bacterial taxa, each employing different mechanisms. These recent examples of the effectiveness of endophytic PGPB in indirectly promoting the growth of different plants are consistent with the successful employment of a wide variety of strategies used by these bacteria in thwarting phytopathogen inhibition of plant growth.

### 2.5. Endophytic PGPB Protect Plants against Abiotic Stresses 

Most of the mechanisms that endophytic PGPB use to promote plant growth help (at least to some extent) to protect plants against various abiotic stresses including high salt, flooding, drought, the presence of inhibitory organic compounds in the soil, and temperature extremes. Since all these abiotic stresses (as well as biotic stresses such as the presence of various phytopathogens) result in the synthesis of growth-inhibiting levels of stress ethylene by the plant subjected to these stresses [45], one of the major mechanisms that endophytic PGPB use to protect stressed plants from abiotic (and biotic) stress is the synthesis of the enzyme ACC deaminase [46]. Moreover, endophytic PGPB that synthesize both ACC deaminase and indole 3-acetic acid are most efficient at enabling plants subject to different types of stress to grow normally.

Recently, several studies have reported that endophytic PGPB with the ability to directly promote plant growth are successful in helping plants to overcome salt stress (a major environmental/abiotic stress worldwide). These reported studies of overcoming salt stress have included tomato [47]; sorghum, cucumber, and tomato [48]; chickpea [49]; and peanut [50]. Moreover, the approach of using endophytic PGPB to overcome abiotic stress has been very recently reviewed [51,52].

## 3. Production of Secondary Metabolites 

### 3.1. Antibiotics 

Human and animal pathogen antibiotic resistance and the emergence of multi-resistant bacterial strains is a current problem of clinical relevance and represents a serious threat to human and animal health worldwide [53]. As a result, there is need to discover new novel antibiotics. Bacterial endophytes are one of the untapped potential sources of novel antibiotics. With high species diversity and adaptation to various environments, endophytes represent a rich source of metabolites [54,55]. Endophytes may have an edge over other microorganisms because of their capacity to defend, communicate with and colonize their plant host, resulting in the production of a large number of structurally diverse secondary metabolites compared with epiphytes or soil microbes [56]. Moreover, because they are symbiotically associated with plants, endophyte-derived antibiotics are likely to be less toxic to humans, which may be of critical importance to the medical community, as potential antibiotics isolated from endophytes may not adversely affect human cells [57]. Antibiotics secreted by endophytes can protect the plant hosts from attack by various phytopathogens [56,58] or prevent insects [59] and nematodes [60] from infecting plants. Other anti-microbial agents are also produced by endophytes that help the host plant to develop systemic resistance against pathogens [61,62]. Additionally, antimicrobials synthesized by microbial endophytes kill or inhibit the growth of plant pathogens including bacteria, fungi, viruses and protozoans that also cause human and animal diseases [63,64]. Some new antibiotics have recently been discovered in endophytes that colonize different plant species [65].

#### 3.1.1. Lipopeptides

Lipopeptides are an important class of secondary metabolites produced by bacterial endophytes and are composed of cyclic or short linear peptides connected to lipophilic molecules. With antibiotic activity against a wide variety of pathogens, these constitute some of the most effective drugs on the market [66]. According to Christina et al. [67], the majority of endophytic bacteria produce lipopeptide antibiotics belonging to three known classes: ecomycins, pseudomycins and kakadumycins. Lipopeptides produced in *Bacillus* and *Paenibacillus* species are well characterized [68]. For example, *B. amyloliquefaciens* and *B. subtilis* are known to synthesize a high level of lipopeptides [69,70]. Interestingly, *B. subtilus* also produces polyketide antibiotics such as bacillomycin, fengycin, iturin, lichensyn, mycosubtilin, plipastin, pumilacidin, and surfactin [70]. Polyketides are small peptide antibiotics that make up a large proportion of industrial antibiotics [71].

For centuries, medicinal plants have been used to cure a plethora of diseases and have more recently been in the spotlight for harboring endophytic microorganisms with rich metabolic potential. For example, many endophytic Actinomycetes found associated with several medicinal plants growing in the Panxi plateau in south-west Sichuan, China produce numerous bioactive molecules with antimicrobial activity against various bacterial pathogens [72]. Endophytic *Actinomycetes* have been isolated from several Chinese medicinal and mangrove plants with antimicrobial activities against the bacterial pathogens *Enterobacter faecalis*, *Staphylococcus aureus*, *Klebsiella pneumoniae*, *Escherichia coli*, *Acinetobacter baumanni*, *Pseudomonas aeroginasa* and some multidrug resistant human pathogens [73]. Another medically important plant native to the Jammu region in northern India was found to be a host to a large number of endophytic bacteria belonging to the genera *Bacillus*, *Pseudomonas*, *Peaenibacillus*, *Acidomonas*, *Streptococcus*, *Ralstonia*, *Micrococcus*, *Staphylococcus* and *Alcaligenes* [74]. Many of the isolates showed antibacterial activity against *B. subtilis* and *K. pneumoniae*. Most of the metabolites from endophytic bacteria were characterized after isolating the bacteria and growing them in vitro. In a recent study on relationships between endophytes and medicinal plants, it was reported that the common bacterial orders associated with these plants were *Bacillales, Enterobacterales*, and *Pseudomonadales*, which accounted for 72.6% of the total isolates [75]. Many species of these genera are of industrial relevance because they produce antibiotics and peptides with anti-microbial, anti-viral and anti-tumor activities [76,77]. According to Beiranvand et al. [78], endophytic *B. thuringiensis* isolated from Iranian medicinal plants produces a broad range of antimicrobial compounds. In addition, Islam et al. [79] isolated *B. thuringiensis* with antibacterial activity from several gymnosperms and angiosperms. Similarly, bacterial endophytes isolated from the leaves of Malaysian and African plants with medicinal properties have been reported to be a rich source of antibiotics exhibiting activities against *S. aureus, B. cereus, E. coli*, and *P. aeruginosa* [80,81].

Antibiotics belonging to kakadumycins, munumbicins xiamycins [82,83,84] and coronomycins [82,85] are predominantly produced by many *Streptomyces* species. Therefore, these species are good candidates for exploring their metabolic potential. *Streptomyces* strains NRRL30566 and GT2002/1503 isolated from the fern-leaved Grevillea tree and mangrove plants, respectively, have produced kakadumycins and xiamycins which have strong antimicrobial activities against several otherwise drug resistant bacteria [73,83]. *Streptomyces* sp. strain SUK06 isolated from the Malaysian medicinal plant *Thottea grandiflora* produced secondary antibacterial metabolites that were effective against some drug resistant strains of *B. cereus, B. subtilus, P. shigelloides, P. aeruginosa* and *S. aureus* [86]. The endophytic strain *Streptomyces* sp. BT01 isolated from the root tissue of the medicinal herb *Boesenbergia rotunda* (L.) has been reported to secrete a rich collection of metabolites [87,88], with strong activities against *B. cereus* and *B. subtilus*. Jasim et al. [89] reported the isolation of *Bacillus mojavensis* from the plant *Bacopa monnieri* produced lipopeptides consisting of fengycin with significant activities against *E. coli, S. aureus, K. pneumoniae*, and *S. typhi*. Fengycin is a cyclic lipopeptide (CLP) produced by *Bacillus* sp. that has potent activity against many antibiotic resistant bacterial strains [90]. Cyclic lipopeptides are often considered to be more attractive than conventional antibiotics because of their unique mode of action [91].

Castillo et al. (82) discovered a novel class of antibiotics called munumbicins (A-D) that have activities against both plant pathogenic fungi and human pathogenic bacteria. These antibiotics are peptide molecules with different ratios of amino acids and were extracted from an endophytic *Streptomyces* strain NRRL 30662 isolated from the stems of the medicinal plant snake vine native to the Northern Territory of Australia. In general, these compounds displayed antibacterial activities against both Gram-positive and Gram-negative bacteria including *B. anthracis*, *S. pneumoniae*, *E. faecalis*, *S. aureus* and multiple drug resistant strains of *Mycobacterium tuberculosis*. Munumbicin D was particularly interesting because it was effective against the malaria causing parasite *Plasmodium falciparum*. These antibiotics appear to be a better alternative to the currently used drug chloroquine because of their higher activity and safety [67]. The same group also isolated another *Streptomyces* sp. 30566 derived kakadumycin that was similar in activity to munumbicins. It was isolated from the fern tree *Grevillea pteridifolia* and exhibited strong bioactivity against many *Bacillus anthracis* strains [83].

#### 3.1.2. Amino Acid-Rich Peptides

*Pseudomonas viridiflava*, a fluorescent bacterium and common leaf endophyte of many grass species, is known to produce ecomycins. These novel lipopeptides are associated with some unusual amino acids such as homoserine and β-hydroxy aspartic acid in addition to common amino acids such as alanine, serine, threonine, and glycine which work against human fungal pathogens [92]. *Pseudomonas syringae*, another endophytic bacterium associated with many plants, can produce pseudomycins, a group of antifungal peptides containing non-traditional amino acids with strong activity against human and plant pathogenic fungi [93].

It can be expected that plants growing in diverse environments are colonized by rare and interesting endophytes with novel bioactive potential. For instance, bacterial endophytes of *Plectranthus tenuiflorus*, a medicinal plant that grows in high altitude and arid environments, displayed strong inhibitory action against many human pathogens including *E. coli*, *K. pneumoniae*, *Proteus mirabilis*, *S. typhi*, *S. aureus*, *Streptococcus agalactiae*, and *Candida albicans* [94]. Moreover, endophytic bacteria from different tissues of the same plant can display different antibiotic resistance profiles and antagonistic interactions. This was the case in a recent study on endophytes isolated from *Echinacea purpurea*, *Echinacea angustifolia* and *Origanum vulgare* [95,96,97]. The authors hypothesize that endophytes may be selected by their antimicrobial resistance phenotypes as a response to antimicrobial metabolites produced by microorganisms in the same niche [96], suggesting that these microorganisms could indeed be a source of new antibiotics and antibiotic resistant mechanisms.

It is possible to discover the genetic characteristics that directly or indirectly control biological functions, as well as putative bioactive secondary metabolites, through genome mining. Genomic characterization of the genera *Bacillus* and *Streptomyces*, well known for synthesizing antimicrobial compounds, indicated the presence of biosynthetic gene cluster (BCGs) such as polyketide synthases (PKSs) and nonribosomal peptide-synthetases (NRPSs) [89]. Among the NRPSmediated products, surfactins, iturins and fengycins from *Bacillus* sp. have been reported for their potent antimicrobial activities [89]. Additionally, bacteria belonging to the genera *Staphylococcus, Micrococcus* and *Sphingomonas* have been shown to have these genes, indicating a more universal distribution of these domains as suitable targets in endophytes where secondary metabolite discovery has substantial potential [98]. Furthermore, characterization of bacterial endophytes of thirty Chinese medicinal herbs on the basis of PKS and NRPS gene clusters suggested the production of known and unknown metabolites with putative bioactivities [99]. These methods offer the additional benefit of rapid screening for biosynthetic pathways involved in secondary metabolism and can be an effective tool as a proxy for investigating endophytes with metabolic potential [99]. Furthermore, bioinformatics tools such as SMURF (Secondary Metabolite Unknown Regions Finder), PRISM (PRediction Informatics for Secondary Metabolomes) and antiSMASH (Antibiotics and Secondary Metabolite Analysis Shell) are helpful in the identification of specific gene clusters involved in the synthesis of bioactive metabolites [99]. 

#### 3.1.3. Cyclic Cationic Lipopeptides 

Polymyxins are also produced by a non-ribosomal peptide synthetase [100]. These antibiotics synthesized by endophytic *Paenibacillus polymyxa* are effective against most members of the *Enterobacteriaceae* family, including *E. coli, Enterobacter* spp., *Klebsiella* spp., *Citrobacter* spp., *Salmonella* spp., and *Shigella* spp. [70]. Examples of NRPS and PKS gene products have been described by Alvin et al. [101]. Recently, four endophytes from the medicinal plant *Origanum vulgare* L. were shown to produce a diverse range of antibiotics including paeninodin, polymyxins and paenicidin. The crude extracts of these endophytes were found to be effective inhibitors against ten strains of a *Burkholderia cepacia* complex known to exacerbate the genetic disease cystic fibrosis. Genomic analysis of the strains revealed the presence of three biogenetic gene clusters (BGCs) including lassopeptide genes, NRP genes and lanthipeptide genes [102].

#### 3.1.4. Pigments as Antibiotics 

Pigments from endophytic bacteria are being explored as sources of new drugs to treat antibiotic resistant pathogens [103]. The pigmented extracts produced by the bacterial endophyte *Burkholderia* sp. WYAT7, isolated from the medicinal plant *Artemisia nilagirica* (Clarke) Pamp., were used as an antibiotic source against several Gram-positive and Gram-negative bacteria. Interestingly, these compounds strongly inhibited the growth of test pathogens including *S. typhi* (MTCC733), *S. aureus* (MTCC1430), *P. aeruginosa* (MTCC2453), *K. pneumoniae* (MTC 432), *E. coli* (MTCC160), *S. paratyphi* (3220), *B. subtilus (441)* and *Acinetobacter baumannii* (12,889), which were obtained from the microbial type culture collection (MTCC) in India [104]. This study provides evidence that bacterial pigments can find applications in pharmaceutical industries. Additionally, biological methods of pigment synthesis provide many advantages over physical and chemical methods by avoiding high energy inputs and the productions of toxic waste, which makes this biological synthesis simple, inexpensive and environmentally friendly.

### 3.2. Anti-Cancer Compounds 

Cancer is a severe disease characterized by uncontrolled cell growth. According to a recent report, cancer is the leading cause of death worldwide, accounting for nearly 10 million deaths in 2020 [105]. The drugs used in the treatment of various cancers show non-specific toxicity for normal cells, have negative side effects, and many are still not active in the treatment of some cancer forms [106]. The discovery of secondary metabolites with cytotoxic properties has provided new insights in anti-cancer treatments [107].

#### 3.2.1. Cyclic Analogs 

Numerous bioactive anti-cancer compounds belonging to different classes such as anthracyclines, glycopeptides, aureolic acids, anthraquinones, enediynes, polysachharides, carzinophilin, mitomycins, alnumycin, pterocidin, napthomycin and alkyl salicylic acids (salaceyins) are reportedly produced by many endophytic bacteria [108]. The anti-cancer potential of endophytic *actinomycetes* bacteria is evidenced in many studies. *Streptomyces* from the Brazilian medicinal plant *Lychnophora ericoides* showed strong cytotoxic activity against human cancer cell lines [109]. The majority of secondary metabolites produced by endophytic bacteria have been characterized after growing them in vitro. Kim et al. [110] grew endophytic *Streptomyces lacey* MS53 in vitro and detected two new anti-cancer agents, salaceyins (A and B), which were cytotoxic to human breast cancer line SKBR3. *Streptomyces* sp. strain DSM11575 isolated from root nodules of *Alnus glutinosa* produced the compound alnumycin, which inhibited growth of K562 human leukemia cells [111]. Studies have shown that acquisition of secondary metabolites with diverse structural compositions from endophytes is affected by the plant’s adaption to a specific niche. This is emphasized by recently described endophytes isolated from plants growing in the tropical wetlands of the Pantanal region of Brazil. Crude extracts of isolates of *Streptomyces albidoflavus* CMRP4852 and *Verrucosispora* sp. CMR P4860 demonstrated anti-melanoma activities with no effect on normal non-cancerous cells [112]. Consequently, the natural products synthesized by endophytic bacteria have attracted enormous interest and research on these strains [113].

The endophytic actinomycete strain YBQ59 isolated from a Chinese cinnamon plant produced metabolites effective against human lung cancer cells [114]. Additionally, Igarishi et al. [115] reported that pterocidins produced by *Streptomyces hygroscopicus* TP-A0451 isolated from *Pteridium aquilinum* exhibited cytotoxic activity to human cancer cell lines NCI-H522, OVCAR-3, SF539, and LOX-IMVI. Similarly, *Streptomyces* sp. CS isolated from *Maytenus hookeri* produced the compound napthomycin, which is effective against P388 and A549 human tumor cells [116]. Sebola et al. [117] tested the anti-cancer activity of crude extracts of bacterial endophytes isolated from *Crinum macowanii* baker bulbs. In this study, the authors observed that *Acinetobacter guillouiae* dramatically reduced growth of the U87MG brain cancer cell line; whereas *Raoultella ornithinolytica* strongly inhibited lung carcinoma cells (62% reduction in cell growth).

#### 3.2.2. Maytansinoids 

Given the role of chemical communication in plants and endophytes, it is understood that certain compounds formerly believed to be synthesized by plants or exclusively considered to be plant metabolites may be produced by endophytes. For instance, Kusari et al. [118] studied the root endophytic communities of *Putterlickia verrucosa* and *Putterlickia retrospinosa* and concluded that maytansine, an anti-cancer agent effective against breast cancer and previously thought to be produced by plants, was in fact synthesized by an endophytic bacterium colonizing the plant roots. Interestingly, the shoot bacterial community did not produce any maytansine. Indeed, the roots may represent a metabolic sink from which to explore bacteria with therapeutic potential. Zhao et al. [119] isolated maytansine producing *Streptomyces* sp. Is9131 from the medicinal plant *Maytenus hookeri.* An extracellular extract of this endophyte was inhibitory to human cell lines implicated in various cancers including leukemia, lung, gastric and liver cancers. Since maytansinoids are an important class of drugs, reportedly more cytotoxic than many anti-cancer drugs, isolation of maytansine-producing bacteria represents an opportunity to discover novel drugs and offers a renewable source of natural products.

#### 3.2.3. Extracellular Metabolites

Exopolysaccharides (EPS) may play a significant role as anti-cancer agents. An endophytic *Bacillus amyloliquefaciens* strain isolated from *Ophiopogon japonicus*, a Chinese medicinal plant, produced EPS that inhibited the growth of human gastric cancer cell lines MC-4 and SGC-7901. EPS-treated cells had abnormal cell morphology and cell death, possibly caused by a mitochondrial dysfunction [120]. This study is a good example of the therapeutic potential of such compounds in anti-cancer applications. Phenolic compounds have also been reported to be involved in various bioactive properties, including anti-cancer activity. For example, two biphenyl producing *Streptomyces* sp. isolated from the root tissue of *Boesenbergia rotunda* (L.) Mansf A. showed strong cytotoxicity against three cancer cell lines (HeLa, HepG2, and Huh7) and less toxicity towards normal cells (L929) [121].

### 3.3. Anti-Viral Compounds 

Since the outbreak of the 2019 coronavirus disease caused by novel coronavirus 2 (SARS-CoV-2), researchers worldwide have been trying to fight the disease with approved drugs or to develop natural anti-coronavirus compounds. 

#### 3.3.1. Flavonoids 

Flavonoids are a large group of bioactive compounds with variable phenolic structures and are synthesized by both plants and microorganisms [122]. Flavonoids such as quercetin, hespertin and naringin show anti-viral activities [123], and apigenin, vitexin, and their derivatives have been shown to be effective against hepatitis C virus, herpes simplex virus 1(HSV-1), human hepatitis A, B, and C, rhesus rotavirus (RRV), and influenza viruses [124]. As a result of the rich metabolic potential of endophytes, there is an emerging interest in the development and use of microbial secondary metabolites as anti-viral agents [125]. To find anti-viral compounds with activities against SARS-CoV2, endophytic bacteria were isolated from various tissues of 16 medicinal plants at the University of Chittagong in Bangladesh. An in vivo study involving extracts of five isolates of endophytes of *Priestia megaterium*, *Staphylococcus caprae*, *Neobacillus drentensis*, *Micrococcus yunnanesis*, and *Sphingomonas paucimobilis*, was carried out to assess their bioactive properties. The highest flavonoid (Quercetin) content was found in the Gram-positive bacterium *S. caprae* with a yield of 45.18 mg/mL. Thus, *S. caprae* may be a potential source of flavonoids for further studies of their anti-viral activity. Additional investigations through molecular docking experiments revealed the presence of two important metabolites, microansamycin and aureusimine, which displayed noteworthy activity against SARS-CoV-2 by altering the viral protease function, thus identifying the possible mode of action by which these extracts can help fight such infections [98].

*Streptomyces* sp. stand out as the most biotechnologically important prokaryotic species that are capable of synthesizing structurally and functionally diverse metabolites. For example, an endophytic Streptomyces from the mangrove tree *Bruguiera gymnorhiza* is the source of a novel anti-HIV compound, xiamycin A [126]. Such studies can pave the way to the development of novel antivirus drugs that might be useful for treatment of HIV infections. 

#### 3.3.2. Saponins

The microbiome of Ginseng plants represents a rich and unique biological niche inhabited by bacteria capable of synthesizing ginsenocides. These compounds include a group of saponins with a triterpenoid dammarane structure produced by the ginseng plants and are highly valuable for their applications in treating a wide variety of medical ailments, including viral infections such as coxsackievirus B3, enterovirus 71, human rhinovirus 3 and haemagglutinating virus of Japan (HVJ) [127]. Moreover, a bacterial endophyte, *Bacillus altitudinis*, has been shown to increase the ginsenoside concentration in the root cultures of *Panax ginseng* [128]. This bacterium is, therefore, a good candidate for further research on its capacity to produce major ginsenosides such as Rb1, Rb2, Rc, Rd, Re and Rg1. In contrast, minor ginsenocides, including compound K, Rg2, Rg3 and Rh2, are more effective and rather rare in the plant host [129]. Interestingly, the rare ginsenocides such as Rh2 and Rg3 are synthesized by bacterial endophytes colonizing *P. ginseng*. These endophytes were identified as β-glucosidase-producing *Burkolderia* sp. GE 17-7 isolated from *P. ginseng* roots [130]. A strategy used to produce the rare ginsenocides included identifying endophytes with the capacity to produce β-glucosidase. A majority of the glucosidase producing strains, including *Arthrobacter* spp., *Enterobacter* spp., *Ochrobactrum* spp., *Serratia* spp., *Burkholderia* spp., and *Flavobacterium* spp., were isolated from *Panax* plants [129]. Therefore, bacterial endophytes have commercial potential in bioproduction and biotransformation of ginsenocides for use as anti-viral agents.

#### 3.3.3. Nanoparticles 

Bacterial cell extracts can be utilized for synthesis of metal-based nanoparticles (NP) which have therapeutic applications. For example, a silver (Ag) resistant *Bacillus safensis* strain TEN12 produced AgNPs intracellularly with a size of 22-42 nm and a spherical shape [131]. Similarly, an endophytic bacterium, *Bacillus cereus*, isolated from the tropical evergreen tree *Garcinia xanthochymus* was shown to synthesize silver nanoparticles [132]. The use of silver nanoparticles has been proposed to treat viral infections caused by HIV-1, hepatitis B virus, respiratory syncytial virus, and herpes simplex virus [133,134,135]. The use of nanoparticles synthesized by bacterial endophytes shows great promise for the development of unique anti-viral compounds.

### 3.4. Other Compounds

#### Terpenoids and Alkaloids

Many plants synthesize alkaloids and terpenoids with wide range of biological properties and many of them have therapeutic effects on human health [55]. Many of these metabolites are also derived from endophytic bacteria. Many medicinal plants host endophytes capable of producing a diverse group of metabolites with high commercial value [3,136]. The ability of these microbes to synthesize secondary metabolites similar to those of their host plants, in some cases even increasing the production of the plants’ secondary metabolites [137,138], provides a fascinating opportunity to explore these excellent resources as new alkaloid and terpenoid producers. Furthermore, when compared with their plant hosts, bacterial endophytes can transform certain alkaloids and terpenoids into more potent and novel derivatives [129].

The bacterial endophyte *Pseudomonas fluorescens* ALEB7B improved the production of sesquiterpenoids in a Chinese medicinal plant [139]. A newly discovered source of camptothecin, a complex pentacyclic pyrroloquinoline alkaloid that is mainly produced and extracted from *Camptotheca acuminata*, a deciduous tree also known as “the Chinese happy tree”, was identified to be the endophytic bacterium *Paenibacillus polymyxa* LY214 [140]. Endophytic bacteria from a medicinal herb used in Chinese medicine were shown to produce guanosine and inosine alkaloid compounds. *Bacillus thuringiensis* and *Bacillus licheniformis* produced the maximum number of alkaloids among the five isolates characterized and tested in this study [141]. Bacteria can stimulate the production of bioactive compounds directly through the modulation of plant gene expression, as demonstrated in the case of benzylisoquinoline (BIA) alkaloids in opium poppy plants inoculated with endophytic *Acinetobacter* SB1B [142]. BIA alkaloids have diverse biological potential as narcotic agents, muscle relaxants and antimicrobials [143]. Analogously, Ptak et al. [144] have shown that *Leucojum aestivum*, a plant with therapeutic properties, inoculated with endophytic *Paenibacillus lautus* isolated from in vitro grown *L. aestivum* plants, had increased levels of galanthamine and lycorine alkaloids. Indeed, this bacterial endophyte is able to modulate the physiology of the plant and its metabolism, as demonstrated by the increased production of indole acetic acid and cytokinins (zeatin and kinetin), gibberellin A, abscisic acid, and salicylic acid, thus providing the precursors (e.g., amino acids) to produce alkaloids. Moreover, the detection of alkaloids such as ismine, lycoramine, haemanthamine, tazettine, galanthamine, lycorine, homolycorine and hippeastrine in the extracts of this bacterium highlights the role of bacterial endophytes in the production of alkaloids for biotechnological and therapeutic applications.

Overall, plant bacterial endophytes as producers of substances of commercial interest appears to be a widespread phenomenon (Table 1).

## 4. Conclusions

In addition to facilitating plant growth and development both directly and indirectly, a number of endophytic PGPB produce a range of secondary metabolites. Many of these secondary metabolites, because of their immense therapeutic value, provide a boon to humanity. Bacterial endophyte-plant interactions offer an example of an ancient, yet ongoing and successful biological partnership that can be exploited to develop, optimize, or increase the production of novel bioactive compounds to be used for their anti-microbial, antiviral, anti-cancer, and anti-inflammatory properties. Technologies to detect endophytic bacteria in vivo from their host and improving culture parameters to obtain metabolites in vitro can further improve the potential value of bacterial endophytes. In addition, bacterial endophytes may be a better choice to derive metabolites than those synthesized from plants because of the perceived benefits such as low costs, a decreased carbon footprint and preservation of plant species.

## Figures and Tables

**Table 1:** Secondary metabolites produced by bacterial endophytes.

Strain Name	Biological Activity	Plant Host	Chemical Class	References
*Pseudomonas viridiflava* EB273	Antifungal	*Lactuca sativa* (lettuce)	Ecomycin	[92]
*Pseudomonas syringae*	Antifungal	*Nicotiana benthamiana* (tobacco)	Pseudomycin	[93]
*Streptomyces* sp.	Antifungal	*Glycine max* (Soybean)	3-acetonylidene-7-prenylindolin-2-one (Alkaloid)	[145]
*Streptomyces* sp.	Antifungal Antitumor	*Allium tuberosum* (Chinese chives)	6-Prenylindole (Alkaloid)	[146]
*Streptomyces* sp. strain NRRL 30562	Antibacterial	*Kennedia nigricans* (Black kennedia)	Munumbicin	[82]
*Streptomyces* sp. NRRL 30566	Antibacterial	*Grevillea pteridifolia* (Darwin silky oak)	Kakadumycin	[83]
*Streptomyces* sp. HK 10595	Antibacterial	*Kandelia candel* (mangrove)	Xiamycin B	[147]
*Aeromicrobium pontii*	Antibacterial	*Vochysia divergens* (Tropical evergreen tree)	1-acetyl-b-carboline (Alkaloid)	[148]
*Actinomycetes*	Antibacterial	Chinese mangrove plants	Erythromycin and levofloxacin-like antibiotics	[73]
*Bacillus* sp.	Antibacterial	*Combretum mole* (medicinal plant)	Flavonoids	[81]
*Streptomyces* sp. MSU-2110	Antibacterial	*Monstera* sp. (tropical plant)	Coronamycins	[85]
*Streptomyces* sp.	Antibacterial	*Alnus glutinosa* alder tree)	Alnumycin	[111]
*Enterobacter* sp. YRL01 *B.subtilis* sp. YRL02	Antibacterial	*Raphanus sativus* L. (Raddish)	Antibiotics	[149]
*Actinomyces*	Antibacterial Antifungal	Chinese medicinal plants	NRPS and PKS	[72]
*Streptomyces parvulus* Av-R5	Antibacterial Antifungal	*Aloe barbadensis miller* (Aloe vera)	Actinomycins	[150]
*Bacillus* sp. 7PJ-16	Antimicrobial	*Morus alba* (Mulberry)	Bacteriocins (Subtilin, subtilosin A)	[151]
*Streptomyces* sp. Is9131	Anti-tuberculosis	*Maytenus hookeri* (medicinal plant)	Maytansine (an ansamycine antibiotic)	[119]
*Kytococcus schroeter*	Anti-cancer	*Ephedra foliate* (Medicinal shrub)	Camptothecin (Alkaloid)	[152]
*Microbacterium* sp. *Burkholderia* sp.	Anti-cancer (Leukemia)	*Coptis teeta * (medicinal herb)	Vindoline (Alkaloid)	[153]
*Bacillus cereus*	Anti-cancer	*Miquelia dentata Bedd. * (Wet forest plant)	Camptothecine	[154]
*Actinomyces* sp.	Anti-cancer	*Bruguiera gymnorrhiza* (mangrove)	Indolocarbazoles (Alkaloid)	[155]
*Streptomyces* sp. YIM66403	Anti-cancer	*Isodon eriocalyx * (medicinal plant)	Anthracyclin	[156]
*Bacillus amyloliquefaciens*	Anti-cancer (gastric)	*Ophiopogon japonicus* (medicinal plant)	Exopolysaccharide	[120]
*Micromonospora lupini*	Anti-cancer(colon)	*Lupinus angustifolius * (Lupin)	Anthroquinones	[157]
*Streptomyces* sp.	Anti-cancer(leukemia)	*Alnus glutinosa* (alder tree)	Alnumycin	[111]
*Streptomyces* sp.	Anti-cancer (lung)	*Maytenus hookeri* (medicinal plant)	Maytansine	[116]
*Streptomyces sp.* strain Is9131	Anti-cancer (gastric, liver, leukemia, lung)	*Maytenus hookeri* (medicinal plant)	Maytansine	[119]
*Streptomyces* sp. BO-07	Anti-cancer (HeLa, HepG2, Huh7 cancer cell lines)	*Boesenbergia rotunda* (medicinal herb)	Biphenyls	[121]
*Streptomyces cavourensis* YBQ59	Anti-cancer (lung)	*Cinnamomum cassia* (medicinal plant)	Bafilomycin D	[114]
*Streptomyces hygroscopicus*	Anti-cancer (NCI-H522, OVCAR-3, SF539, LoX-IMVI cell lines)	Herbaceus plants	Pterocidin	[115]
*Streptomyces laceyi* MS53	Anti-cancer(breast)	*Ricinus communis* (Castor plant)	Salaceyins A, B	[110]
*Burkholderia* sp.	Anti-cancer	*Panax ginseng* (Asian ginseng)	Ginsenoside Rg3 (Saponin)	[146]
*Streptomyces* sp. GT2002/1503	Anti-HIV	*Bruguiera gymnorrhiza* (mangrove)	Xiamycin A	[126]

## Data Availability

Not applicable.
